# Effect of age on the mercury sensitivity of zebrafish (*Danio rerio*) sperm

**DOI:** 10.1007/s10695-020-00875-9

**Published:** 2020-09-17

**Authors:** Bernadett Pataki, Berta Izabella Roberta, Gyöngyi Gazsi, Béla Urbányi, Tímea Kollár, Ákos Horváth

**Affiliations:** Department of Aquaculture, Szent István University, Páter Károly u. 1, Gödöllő, H-2100 Hungary

**Keywords:** Zebrafish sperm, Mercury toxicity, CASA, Male age

## Abstract

The effect of age on the sensitivity of zebrafish sperm against mercury exposure was investigated in the present study. Although results of the use of sperm from mature individuals for toxicity tests have been published, there is no information about the exact age of the fish in some cases, which can affect the results. During the experiments, pooled sperm was stripped from males of 7, 12, or 18 months of age, divided into 5 sub-groups, diluted with different concentrations of Hg (0, 0.5, 1, 2.5, and 5 mg/L Hg), and incubated for 240 min. The motility parameters of sperm (progressive motility (%), curvilinear velocity (VCL)) were measured by a computer-assisted sperm analysis system, at 30, 120, and 240 min of exposure. Regarding the age, significant differences were found in PMOT (*p* = 0.0267) as well as in VCL (*p* = 0.0004) among the three different age groups. The different concentrations of Hg also caused significant differences. The most significant differences in PMOT were between the 7- and 18-month-old groups; these differences were observed at 0.5, 1 and 2.5 mg/L Hg at 30 min, at 0.5 and 1 mg/L at 120 min, as well as at 0.5 mg/L at 240 min. In VCL the most significant differences were found between the 7- and 12-month-old groups; significant differences were found at each tested concentration at 30 min as well as at 0.5 and 2.5 mg/L at 240 min. According to the results, the age of zebrafish negatively influences the sensitivity of its sperm. This may concern not only toxicology tests but many techniques in fish breeding where the sperm is treated before use (cryopreservation, pressure shock, etc.).

## Introduction

The effect of male aging on sperm quality can vary among species; some species react more sensitively, while others do not have any effect on sperm characteristics regarding aging. In the case of human sperm examined with computer-assisted sperm analysis (CASA), sperm parameters decline with age (Fréour et al. 2011), and it is the same in the case of brown Norway rat (*Rattus norvegicus*) sperm (Syntin and Robaire 2001). On the other hand, Martínez et al. (2008) have not found any significant difference in sperm quality parameters measured with CASA between the age groups (1.5–7.5 years) in Iberian red deer (*Cervus elaphus hispanicus*).

Less information is available on the effects of aging on the characteristics of teleost gametes. Regarding sperm characteristics, parameters provided by CASA such as progressive motility and curvilinear velocity are the most important ones, because they are the most sensitive; they react earliest to toxic exposure (Kollár et al. 2018ab). In the previous studies, sperm swimming velocity increased parallel with age in bluegill (*Lepomis macrochirus*) (Casselman and Montgomerie 2004), whereas aging in the guppy (*Poecilia reticulata*) is associated with altered morphology and cell concentration (Gasparini et al. 2010). On the other hand, it causes an increase in sperm production, in body coloration, and in sexual behavior in guppy males (Evans et al. 2002). In the case of striped bass (*Morone saxatilis*), 3-year-old fish produced the greatest number of spermatozoa, had significantly higher percentage of motile sperm, and had higher motility time in comparison to younger or older fish. The authors also concluded that the short-term storage might be affected by the age of the male fish (Vuthiphandchai and Zohar 1999). In another study working with pejerrey (*Odontesthes bonariensis*) using CASA, Chalde et al. (2014) found no significant difference between the 3-, 5-, 7-, and 10-year-old groups in the sperm quality. Similarly, testing the fertility rate in salmon (*Oncorhynchus nerka*), Hoysak et al. (2004) found no difference between the age groups (3, 4, 5 years). In the Atlantic salmon (*Salmo salar*), precociously maturing farm-raised parr had higher fertilization success and greater ATP levels in a sperm competition situation than mature anadromous males (Vladić et al. 2010). In the case of zebrafish, Diogo et al. (2018) found that motility decreases with age; however, the zebrafish line itself had the largest main effect on motility. Kanuga et al. (2011) found no significant difference between the sperm motility of the young and old groups of zebrafish. Johnson et al. (2018) were studying the motility and velocity parameter changes with CASA in old and young zebrafish and found that curvilinear velocity (VCL) significantly dropped after 4 months of age. In zebrafish, age-related phenotypic changes and the appearance of a senescence-associated β-galactosidase staining were described in previous studies (Gerhard and Cheng 2002; Gerhard et al. 2002, Gerhard 2007; Kishi et al. 2003; Keller and Murtha 2004).

It is not rare to see an increase in the fertilization rate, sperm morphology, or progressive motility with age in case of fish (Vuthiphandchai and Zohar 1999; Hoysak et al., 2004; Gasparini et al. 2010; Gilbert et al. 2014), although these results are in contrast with results in mammals or other species (Service PM and Fales 1993; Uglem et al. 2001; Schafer & Uhl, 2002; Radwan et al. 2005; Jones et al. 2007). These differences can be linked to milt volume. In case of the rainbow trout (*Oncorhynchus mykiss*), the mean milt volume was 3 times higher at the age of 3 years than at the age of 2 years (Büyükhatipoglu and Holtz 1984). Unlike humans, fish grow during their life, and the amount of semen should be shown in respect of weight ratio (Kidd et al. 2001).

In toxicology studies involving heavy metals both embryos and adult zebrafish are used (Dave 1985; Roales and Perlmutter 1974). Heavy metals can either have effects on the development of embryos (Hassan et al. 2012; Zhu and Shi 2002) or can inhibit the activity of enzymes (Richetti et al. 2011; Senger et al. 2010) and can even affect gene expression (Wu et al. 2012). Zebrafish can also be used for monitoring Hg^2+^ and Cd^2+^ levels in water through measurement of mRNA levels (Chan et al. 2006).

Mercury is known to be one of the most toxic heavy metals (O’Shea 1999). It occurs naturally in the environment (soil, water, air) which means that every living organism is exposed to a low level. Usually, rocks and soils contain 1 or less than 1 ppb of mercury. Surface waters contain less than 0.1 ppb, while the atmosphere holds 16 ppb of mercury (Hammond 1971). The most common forms of mercury in the environment are metallic mercury, mercury sulfide, mercuric chloride, and methylmercury. From these forms, methylmercury is able to bioaccumulate and biomagnify in fish and also in marine mammals (Fitzgerald et al. 2005, 2007). In older and predatory fish, mercury accumulates primarily in the liver, kidneys, brain, and muscle. In the case of humans as well as in animals, it can be nephrotoxic, immunotoxic, neurotoxic, and mutagenetic (O’Shea 1999). The effect of mercury on sperm quality has been investigated in case of many fish species. In these studies, reduced motility parameters were observed followed mercury exposure in case of rainbow trout (*Oncorhynchus mykiss*; Dietrich et al. 2010), common carp (*Cyprinus carpio*; Chyb et al. 2001a, b), African catfish (*Clarias gariepinus*; Lahnsteiner et al. 2004), Eurasian perch (*Perca fluviatilis*; Hatef et al. 2011), and zebrafish (*Danio rerio*; Kollár et al. 2018a). Furthermore, the antioxidant enzyme activity was affected in case of common carp (Sarosiek et al. 2009), as well as the fertilization rate was affected in case of African catfish (Rurangwa et al. 1998). However, the effect of aging on sperm sensitivity is a crucial information as gamete quality (including morphology, motility features) affects the fertilizing capacity of sperm (Kime et al. 2001; Rurangwa et al. 2004; Alavi and Cosson 2005); thus, gametes with reduced quality can lead to reduced fertilization rate. Despite of this, it was not examined in the previously mentioned studies regarding the effect of mercury on fish sperm quality.

There are many studies on the use of fish sperm as an in vitro toxicology model (Chyb et al. 2000; Chyb et al. 2001a, b; Hatef et al. 2010; Gazo et al. 2013; Kollár et al. 2018a, b). In these, the main goal was either to monitor the effects of heavy metals on reproduction or to offer an in vitro test for measuring toxicity. Most of them concluded that heavy metals decrease the quantity of sperm. However, in toxicology tests involving sperm, the age of male individuals is typically unknown; the fish are simply considered mature. Thus, it is important to know if the difference in age can affect motility parameters. Furthermore, this is also relevant in environmental conditions: fish are externally fertilizing species; thus, during fertilization, gametes can get in direct contact with toxicants present in water. Consequently, the sperm of aged fish can react more sensitively to this exposure which can lead to reduced fertilizing ability. Therefore, in this study, we investigate the effect of male aging on sperm susceptibility to mercury exposure.

## Materials and methods

### Broodstock handling

Sexually mature wild-type males of zebrafish (*Danio rerio*) at 7, 12, and 18 months of age were selected for sperm collection (zebrafish are the most active sexually in this range of age). The fish were kept in 3-L polycarbonate tanks at 25 ± 2 °C (pH 7.0 ± 0.2; conductivity 525 ± 50 μS; alkalinity 0 mM OH^−^, 0 mM CO_3_^2−^, and 0.4 mM HCO_3_^2−^; hardness < 0.5 ^o^dH; DOC > 90%; from here onwards referred to as system water) in a recirculating zebrafish housing system (ZebTEC® (Tecniplast, Italy)) at the Department of Aquaculture, Szent István University (Gödöllő, Hungary). The photoperiod was 14-h light and 10-h dark. Fish were fed twice a day with commercial zebrafish feed (ZEBRAFEED® diet (Sparos Lda, Portugal) and with live artemia (*Artemia salina* nauplii) (TQ type; INVE Aquaculture NV, Belgium) every other day.

### Collection of sperm

Firstly, the fish were anesthetized with tricaine methane sulfonate (MS222 Arlos Organics™, Geel, Belgium, 168 mg/L). After the anesthesia, the sperm was collected by stripping of males as follows. The fish were placed into a dampened foam holder, and sperm from the males were collected into 10-μL glass capillaries by abdominal massage of the individuals using a slide forceps. From each age group, 5 μL of semen was pooled directly into 25 μL of cyprinid immobilizing solution (200-mM KCl, 30-mM Tris, pH 8; Saad and Billard 1987). This quantity was collected from 9 males (*n* = 9; 3 age groups × 3 males). The samples were stored on crushed ice until and during further measurements. After the collection of sperm, fish were recovered from anesthesia and replaced into their housing tank.

### Sperm dilution and exposure

The stock solution was prepared by dissolving mercury (II) nitrate monohydrate (Hg(NO_3_)_2_ × H_2_O, Sigma Aldrich, St. Louis, USA) in a cyprinid immobilizing solution (200-mM KCl, 30-mM Tris, pH 8; Saad and Billard 1987) and was stored at − 80 °C up to 7 days. The final solutions from the stock were prepared immediately prior to the experiments. Sperm pools from a given age group (*n* = 3) were divided into 5 sub-groups (5 μL/each), and each sub-group was exposed to 5 μL of double-concentrated Hg solution in order to reach the final concentration of the tested chemical (0, 0.5, 1, 2.5, and 5 mg/L Hg). Exposure concentrations refer to immediate concentrations of Hg ion. The incubation time was 240 min in PCR tubes (Eppendorf™, 0.2 mL) on crushed ice.

### Sperm motility analysis

Sperm quality was measured using a computer-assisted sperm analysis (CASA) system (Sperm VisionTM v. 3.7.4., Minitube of America, Verona, USA) including an Olympus BX 41 microscope with a negative-phase contrast objective (× 20 magnification). Progressive sperm motility (PMOT, the percentage of cells performing forward movement, %; WHO 2010) and curvilinear velocity (VCL, time-averaged velocity of a sperm head along its actual curvilinear path, μm/s WHO 2010) were measured at 30th, 120th, and 240th minutes of exposure. The samples were activated in a Makler chamber (SEFI Medical Instruments, Haifa, Israel) with system water supplemented with 2% bovine serum albumin (BSA) in a 1:5 dilution ratio in the case of fresh sample to check the quality. Only sperm samples with PMOT higher than 60% were used in the experiments. In case of diluted samples, a dilution ratio of 1:2.5 was used for activation. Activation was carried out two times in case of all samples. The activating solution was stored on crushed ice as well; thus, its temperature was the same as of the samples. The number of detected spermatozoa was between 100 and 500 per field, in order to improve the accuracy of the analysis. Motility was recorded within 5 s after activation.

### Statistical analysis

Statistical analysis was performed using the software GraphPad Prism 6.0 for Windows. Two-way ANOVA with Tukey’s post hoc test was used to determine the main effects of age and Hg concentrations on the PMOT and VCL values of the sperm at different measuring points in time. These motility parameters were chosen according to a previously published study investigating the effect of mercury on zebrafish sperm (Kollár et al. 2018a). The significance level was *p* = 0.05 in each measurement.

## Results

**Fig. 1:**
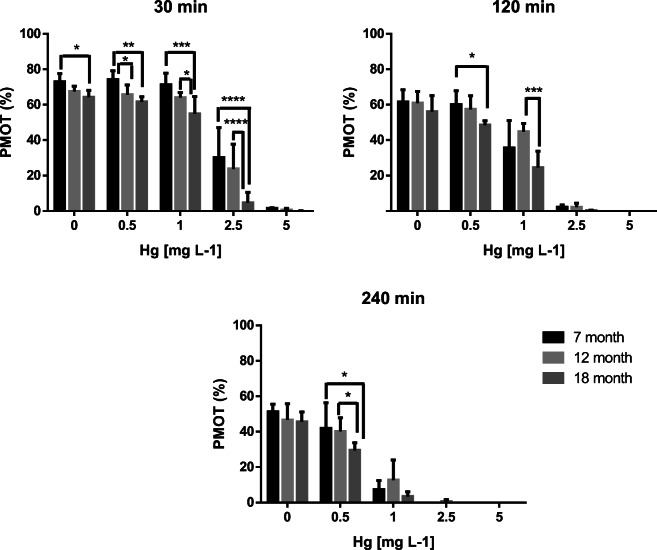
Average progressive motility (PMOT) with SD (*n* = 3) of zebrafish sperm exposed to various Hg concentrations recorded at the 30th, 120th, and 240th minute of exposure. Significant differences among the tested groups of age are labeled with an asterisk (*) at *p < 0.05, **p < 0.01, ***p < 0.001, and ****p < 0.0001

Regarding PMOT, no interaction of Hg concentrations and fish age (*p* = 0.3038) was detected at 30 min of exposure. Sperm PMOT, however, was affected by the Hg concentrations (*p* < 0.0001) as well as by the age of fish (*p* = 0.0267) (Fig.1). A significant difference (*p* < 0.05) was found between the PMOT of the untreated (0 mg/L) groups aged 7 (73 ± 5%) and 18 months (64 ± 4%). At 0.5 mg/L, the 7-month-old group showed significantly higher PMOT value (74 ± 5%) than the 12- (66 ± 5%; *p* < 0.05) or 18-month-old (62 ± 3%; *p* < 0.01) groups. At 1 mg/L dose of mercury, significant differences were found between PMOT values of 7- (71 ± 7%) and 18-month-old (55 ± 10%) as well as 12- (64 ± 3%) and 18-month-old fish. At 2.5 mg/L, these differences were even higher (*p* < 0.0001) in both cases. The PMOT of the youngest group was 30 ± 17%, while it was 24 ± 14% in the 12-month-old group and 5 ± 6% in the18-month-old group. At 5 mg/L, mercury reduced the progressive motility to the extent that no significant differences in PMOT were detected among the different age groups of zebrafish (1 ± 0%, 1 ± 1%, and 0 ± 0%, respectively). At 120 min of exposure, only the concentration of mercury had a significant effect (*p* < 0.0001) on PMOT. Accordingly, there was no significant difference among the groups of age in the untreated (0 mg/L) zebrafish. Furthermore, significant differences were found in 2 cases: one at 0.5 mg/L between the 7-month-old (60 ± 8%) and 18-month-old fish (49 ± 2%; *p* < 0.05) and the other at 1 mg/L of mercury between the 12-month-old (36 ± 15%) and 18-month-old fish (25 ± 9%; *p* < 0.001). At 240 min of exposure, again only the concentration had a significant effect on PMOT (*p* < 0.0001). No significant differences were found among the age groups in the untreated (0 mg/L) individuals. Only in case of 0.5 mg/L was a significant difference found: PMOT of the 18-month-old group (30 ± 4%) differed significantly from that of the 7- (42 ± 14%; *p* < 0.05) and the 12-month-old group (40 ± 8%; *p* < 0.05). In most of the cases, the increase of Hg concentration as well as the aging had a negative effect on PMOT.

**Fig. 2:**
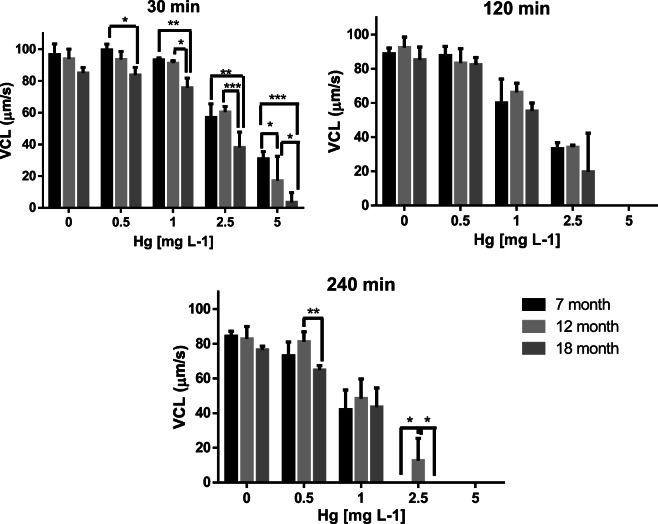
Average curvilinear velocity (VCL) with SD (*n* = 3) of zebrafish sperm exposed to various Hg concentrations recorded at the 30th, 120th, and 240th minute of exposure. Significant differences among the tested groups of age are labeled with an asterisk (*) at *p < 0.05, **p < 0.01, and ***p < 0.001

Similarly to PMOT, in the case of VCL at 30 min of exposure, no interaction of Hg concentration and fish age (*p* = 0.2607) was observed (Fig.2). On the other hand, significant main effects of concentration (*p* < 0.0001) as well as age (7, 12, or 18 months) (*p* = 0.0004) on VCL were found. At this point of time, no significant difference was found in the untreated (0 mg/L) group; however, at 0.5 mg/L Hg concentration, a significant reduction was observed between the 7- (99 ± 4 μm/s) and the 18-month-old fish (85 ± 3 μm/s; *p* < 0.05). At 1 mg/L, the average curvilinear velocity of the 18-month-old group (76 ± 6 μm/s) was significantly different from the 7-month-old group (93 ± 1 μm/s; *p* < 0.01) and from the 12-month-old group (91 ± 1 μm/s; *p* < 0.05). At 2.5 mg/L of mercury, the VCL of 18-month-old group (38 ± 10 μm/s) was significantly lower than that of the 7- (57 ± 8 μm/s; p < 0.01) and 12-month-old groups (60 ± 3 μm/s; *p* < 0.001). At 5 mg/L, all the groups differed significantly from each other. The VCL value was 31 ± 4 μm/s in the 7-month-old group, while in the 12-month-old group, it was 17 ± 15 μm/s and 4 ± 6 μm/s in the 18-month-old group. At 120 min of exposure, only the Hg concentration had a significant negative effect on VCL (*p* < 0.0001); however, there was no significant difference among the different age groups, neither in the case of untreated (0 mg/L) group. In the case of 240 min of exposure, concentration (*p* < 0.0001) and age (*p* < 0.05) had a significant effect on VCL values, again, without significant interaction (*p* = 0.0923). Again, no significant differences were found among the different groups of age in case of the untreated males (0 mg/L). However, at the dose of 0.5 mg/L Hg, a significant difference between the 12- (40 ± 8 μm/s) and the 18-month-old fish (30 ± 4 μm/s; *p* < 0.01) was found. Furthermore, in case of 2.5 mg/L exposure, the 12-month-old group (1 ± 1 μm/s) differed significantly from the 7- as well as from the 18-month-old group: in both groups, the VCL was 0 ± 0 μm/s (*p* < 0.05 in both cases). In most of the cases, the increased Hg concentration as well as the aging had a negative effect on VCL.

## Discussion

During our experiments, our hypothesis that exposure of mercury triggers a dose response in progressive motility and average curvilinear velocity parameters of the sperm of aging zebrafish and that the effective concentrations reduce in parallel with aging was found to be correct. In case of the bluegill, Casselman and Montgomerie (2004) found that the sperm swimming speed increased parallel with age (*p* = 0.047). However, in our study, the opposite was found in the case of the zebrafish, as velocity reduced significantly (*p* < 0.05) among the different age (8, 12, 18 months) groups. In case of the striped bass, Vuthiphandchai and Zohar (1999) were working with 1-, 3-, and 12-year-old individuals. The sperm was stored at 4 °C, and the sperm of 3-year-old fish was found to have significantly (*p* < 0.05) higher motility than the other groups. In our study, not just age but mercury also had a negative impact on zebrafish sperm motility; the sperm of 7- and 12-month-old individuals was more resistant to the toxicant than that of 18-month-old group.

In the study of Chalde et al. (2014), no significant difference (*p* > 0.05) was found between the motility parameters of differently aged pejerrey groups. They were working with 3-, 5-, 7-, and 10-year-old fish where not all the 3- and 10-year-old fish were able to be stripped, and some individuals of 3-year- old group were not subjected to CASA due to insufficient quantity (Chalde et al. 2014). However, in our research, all the zebrafish males were able to give sperm, and a significant (*p* < 0.05) difference in PMOT was found among the different aged groups in the non-treated groups at 30 min. This difference increased when sperm was exposed in mercury. There were no significant differences in PMOT with time in case of the different untreated groups, and the difference was reduced in the mercury-exposed groups as well. In the case of VCL, there was no significant difference in the different untreated groups at any exposure time, and the difference was the highest at 30 min in the exposed groups. As in case of PMOT, the time-dependent difference was reduced in the mercury-exposed groups.

Johnson et al. (2018) studied VCL and motility parameters correlated with age in zebrafish. They found that after the age of 4 months VCL significantly reduced from 120 to 90 μm s^−1^ at the age of 31 months. Motility remained around 90% until the age of 22 months and suddenly decreased after that age (Johnson et al. 2018). Although no significant (*p* = 0.147) age-related decrease of VCL was found in our study (the oldest fish were 18 months old), a significant difference (*p* < 0.001) was observed in the non-exposed groups in the case of PMOT between the 8- and 18-month-old fish.

Diogo et al. (2018) have investigated the sperm of 6-, 8-, 12-, and 14-month-old zebrafish. It was found that the 8-month-old individuals have significantly (*p* < 0.001) higher motility than the 12- and 18-month-old groups. In our research we found the same significant difference (*p* < 0.05) in case of the untreated group between 7- and 18-month-old fish after 30 min of storage. Contrary to our results and those of Diogo et al. (2018), Kanuga et al. (2011) found no significant difference (*p* > 0.05) between the sperm motility of young and old zebrafish males at 10, 30, 20, 120, 180, 300, and 420 s post activation. They found that there was no significant difference neither in the percent motility (*p* = 0.648) nor in the velocity (*p* = 0.535).

In this study, a time-dependent dose response was observed in different age groups of zebrafish. In the different points of time, the sensitivity of the sperm from different age groups of males was not uniform: At one point the youngest group proved to be the most resistant (e.g., 120-min exposure, 0.5 mg/L group), while in another measuring point, the middle-aged group was the least sensitive (e.g., 120-min exposure, 1 mg/L group). There is no exact tendency in the change of motility parameters affected by the age. However, the simple fact that there is a difference in motility parameters among the different aged groups affected by mercury exposure is a crucial information, because in most studies (e.g., in toxicological-aimed studies or in cryopreservation), this important factor is ignored. These results show that in sperm toxicity studies, the age of males has to be considered. This can also be problematic in environmental conditions where fish are exposed to toxicants during spawning. This can lead to impaired fertilization which has a stronger effect in aged fish. These observations allow us to conduct further studies to concentrate on the effect of other toxic substances and even on sperm cryopreservation of aged zebrafish.

## Conclusions

Zebrafish is an appropriate donor for the spermatoxicological experiments in which the most important is to avoid intrinsic factors affecting sperm motility. In this research, we observed that the age of zebrafish is also a factor that can influence the sensitivity of sperm which concerns not only toxicology tests but many techniques in fish breeding in which sperm is treated before use (cryopreservation, pressure shock, etc.). Furthermore, it can be problematic in case of environmental circumstances, during the fertilization of aged fish.
